# AFM13: a first-in-class tetravalent bispecific anti-CD30/CD16A antibody for NK cell-mediated immunotherapy

**DOI:** 10.1186/s13045-015-0188-3

**Published:** 2015-08-01

**Authors:** Jingjing Wu, Jiaping Fu, Mingzhi Zhang, Delong Liu

**Affiliations:** Department of Oncology, The First Affiliated Hospital of Zhengzhou University, Zhengzhou, 450052 China; Department of Hematology, Shaoxing People’s Hospital, Shaoxing, Zhejiang Province China; Division of Hematology & Oncology, New York Medical College, Valhalla, NY 10595 USA

## Abstract

Monoclonal antibodies against CD20 molecule have been leading the revolution of lymphoma treatment. In addition to monoclonal antibodies against CD20 and CD30, novel agents of immunotherapeutics in clinical development are being developed and are rapidly migrating to clinical application. One area of active development is NK cell activators, such as AFM13. This review will highlight the latest development of AFM13 as the first-in-class tetravalent bispecific anti-CD30/CD16A antibody for NK cell-mediated immunotherapy.

Precision therapy with targeted agents against tyrosine kinases and cancer-associated molecules has been leading the revolution of cancer treatment [1–7]. Cancer immunotherapy represents another wave of revolution in cancer therapy [8–13]. In addition to monoclonal antibodies against CD20 and CD30 [14, 15], the main agents of immunotherapeutics in clinical development include the following : (1) immunomodulators (e.g., lenalidomide) [16]; (2) immune checkpoint blockers (e.g., pembrolizumab, nivolumab, ipilumumab) [10, 11, 17]; (3) T cell activators (e.g., CAR-T19; blinatumomab, AFM11) [18–20]; (4) inhibitors of B cell receptor signaling (e.g., ibrutinib) [2, 21]; and (5) NK cell activators (e.g., AFM13) [22]. AFM13 is a first-in-class tetravalent bispecific anti-CD30/CD16A antibody for NK cell-mediated immunotherapy.

## NK cell-activating bispecific antibody (bsAb)

CD16 (FcγRIII) is a low-affinity receptor for the IgG Fc domain and has two isoforms, CD16A and CD16B [23]. CD16A is an activating receptor mainly expressed on NK cells and macrophages. CD16B is expressed mainly on granulocytes and is not involved in tumor cell killing [23]. CD30 is expressed mainly by the Hodgkin and Reed-Sternberg cells in patients with Hodgkin’s lymphoma (HL). A bispecific antibody against CD30/CD16, HRS-3/A9, was reported to bind to the CD30 antigen with one arm, whereas the other arm binds to the CD16 antigen [24]. This HRS-3/A9 bsAb was shown to recruit and activate NK cells and induce complete remission of CD30+ tumors [24]. Phase I/II studies were done in 15 patients with refractory HL [25, 26]. HRS-3/A9 was infused every 3 to 4 days for a total of 4 times, starting with 1 mg/m^2^. The maximum tolerated dose (MTD) was not reached at 64 mg/m^2^, the highest dose administered, because of the limited availability of HRS-3/A9. Nine of the 15 patients developed human anti-mouse Ig antibodies. Four of the patients had an allergic reaction on retreatment. One complete remission (CR) and one partial remission (PR) were seen. These studies led to the further development of NK-activating bsAbs.

## AFM13

**Fig. 1:**
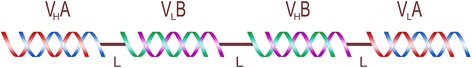
Gene structure of tetravalent bispecific AFM13 antibody domains. The heavy and light chain DNA sequences of CD30 and CD16A were molecularly engineered in the special order as shown. This figure was modified from Rothe et al. and Reusch et al. [22,27]

**Fig. 2:**
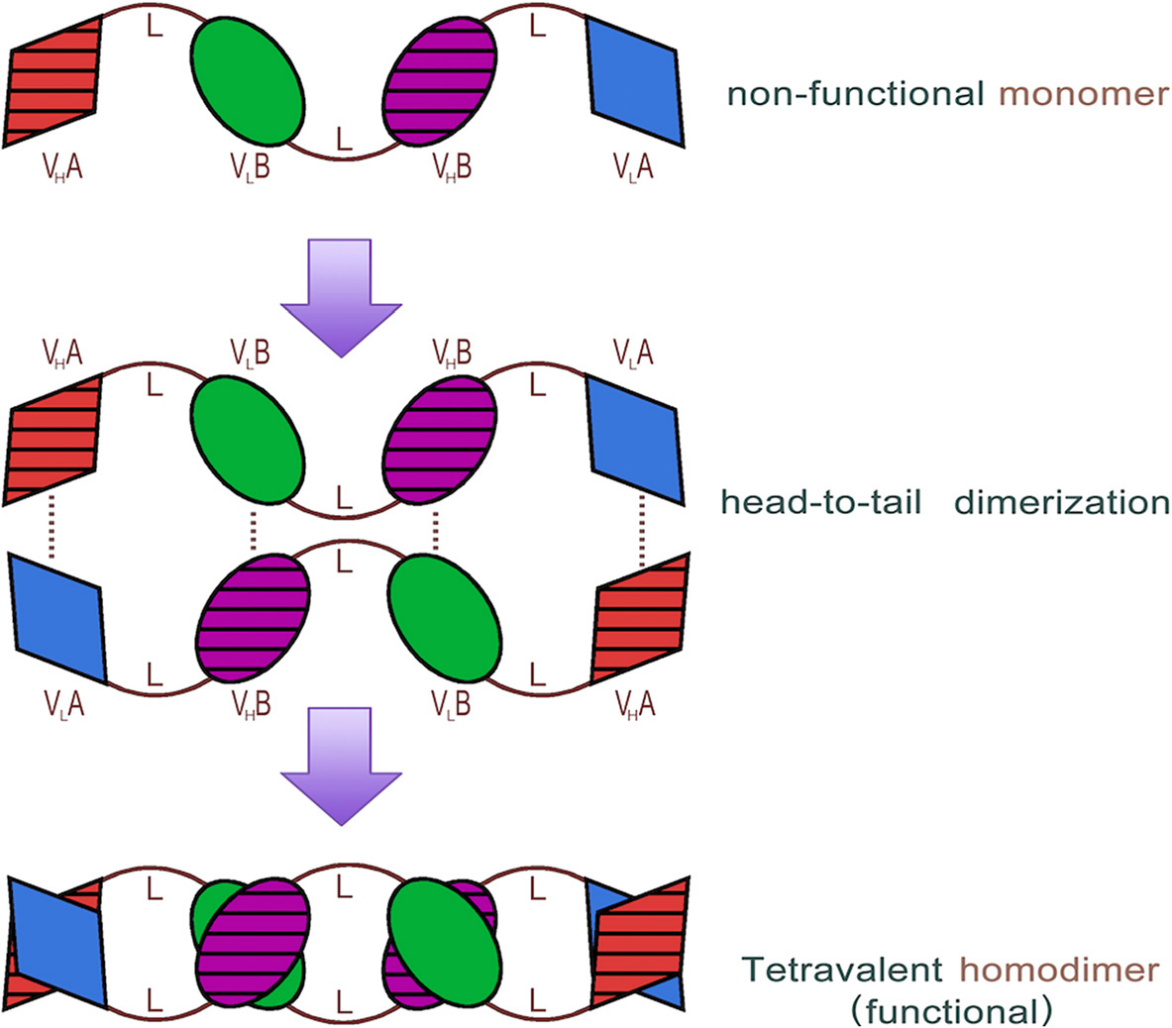
Protein structure and antibody formation pathway of the tetravalent bispecific AFM13 antibody. The CD16A (domain A, *diamond shape*) and CD30 (domain B, *oval shape*) peptide domains were linked by a 9-amino acid linker (*L*) to form a single polypeptide (nonfunctional monomer). A fully functional tetravalent bispecific chimeric antibody construct (*TandAb*) is formed by homodimerization of the single polypeptide monomer in a head-to-tail fashion through non-covalent interactions (*dotted lines*) of the domains in the Ig heavy (*V*_H_) and light (*V*_L_) variable chains. The AFM13 TandAb has a molecular weight of approximately 104 kDa. This figure was modified from Rothe et al. and Reusch et al. [22, 27]

**Fig. 3:**
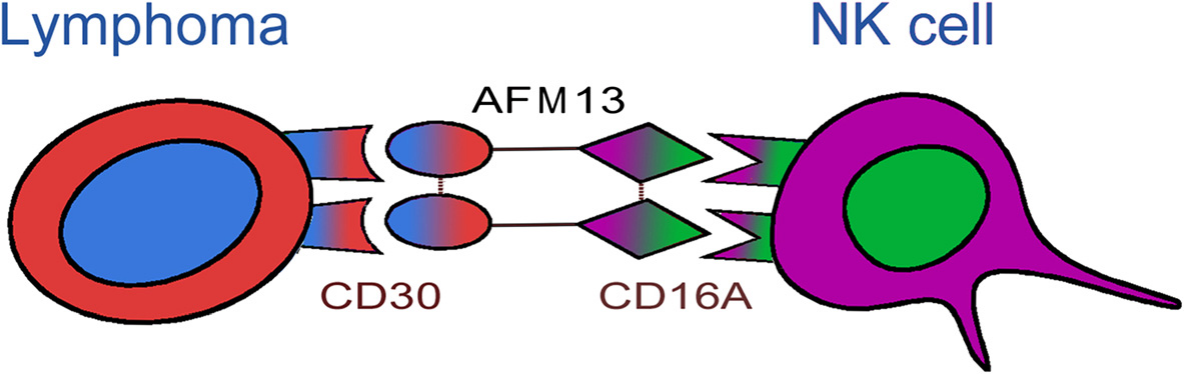
AFM13-mediated activation of NK cells. One arm of AFM13 binds to the CD30 antigen on lymphoma cells, whereas the other arm binds to the CD16A antigen on the NK cells. The activated NK cells destroy the lymphoma cells. The NK cell activation and lymphoma destruction mediated by AFM13 are CD30-dependent

AFM13 is a tetravalent bsAb against CD30 and CD16A produced from the mammalian CHO cells by Reusch et al. [27]. Initially, a human anti-CD16A antibody with no binding to 16B isoform was isolated. The variable anti-CD16A-specific human scFv was then derived. The anti-CD30 Fv domain was derived from the murine HRS-3 IgG. The heavy and light chain DNA sequences of CD30 and CD16A were then molecularly engineered in a special order (Fig. 1) [27]. The CD30 and CD16A peptide domains were linked by a 9-amino acid linker peptide to form a bispecific diabody [28]. A tandem diabody with four domains was engineered to form a single polypeptide (nonfunctional monomer) (Fig. 2). A fully functional tetravalent bispecific chimeric antibody construct (TandAb) is formed by homodimerization of the single polypeptide monomer through non-covalent interactions of the domains in the Ig heavy (*V*_H_) and light (*V*_L_) variable chains. The TandAb has a molecular weight of 104 kDa. One arm of AFM13 binds to the CD30 antigen on lymphoma cells, whereas the other arm binds to the CD16A antigen on the NK cells (Fig. 3). The anti-CD30/CD16A tetravalent bsAb AFM13 was shown to have an IC_50_ value of 35.8 nM for CD30 antigen. Cytotoxicity assays showed that the AFM13-mediated activation of NK cells was strictly CD30-dependent. In the absence of CD30 target cells, neither cytotoxicity nor NK cell activation was elicited by the TandAb [27].

AFM13 was studied by the groups in Germany and in MD Anderson Cancer Center in a phase I dose-escalation study in 28 patients who have been heavily pretreated for their relapsed/refractory CD30+ HL (AFM13-101, NCT01221571) [22]. AFM13 was infused weekly for 4 weeks as one cycle at doses ranged between 0.01 and 7 mg/kg body weight. The MTD was not reached. The only dose-limiting toxicity reported in the study was hemolytic anemia in a patient who received 3 infusions at 0.5 mg/kg. Significant NK cell activation and reduction of soluble CD30 in peripheral blood were reported, though the best clinical response was only PR (11.5 %, 3/26 evaluable patients). In patients who received AFM13 at a dose of ≥1.5 mg/kg, the overall response rate was 23 % and the overall disease control rate was 77 % in the heavily pretreated subjects. Brentuximab vedotin (BV) is an antibody-drug conjugate which also binds to CD30 and delivers a chemotherapeutic agent to the CD30+ cells [15]. In this study, AFM13 was found to be active in BV-refractory patients. Since the effector mechanism of these two antibodies is entirely different, AFM13 represents a novel agent for HL refractory to BV.

## Issues and future directions

AFM13 is a chimeric antibody with a murine anti-CD30 domain. Antidrug antibodies (ADA) were detected in 15 out of 28 patients. Half of the detected ADAs had neutralizing potential. This remains an issue for further investigation in a large cohort. The half-life of AFM13 was 19 h, which is longer than that of the smaller bispecific BiTE antibody, blinatumomab, which is administered as a continuous infusion [19, 29]. Dosage, dosing-schedules, such as twice-weekly dosing, and duration of treatment are the issues for further investigations. Future studies are also needed for correlation of clinical activity with biomarkers, such as NK cell numbers, and soluble CD30 molecules. Evaluation of these biomarkers in the biopsy specimens will also be informative. A phase II study is underway for this novel tetravalent bsAb AFM13 as the first-in-class NK cell-specific agent for cellular immunotherapy targeting CD30+ malignancies (GHSG-AFM13, NCT02321592). Additional tetravalent bsAbs are also being engineered for targeting other malignancies [20, 30].

## References

[1] Akinleye A, Avvaru P, Furqan M, Song Y, Liu D (2013). Phosphatidylinositol 3-kinase (PI3K) inhibitors as cancer therapeutics. J Hematol Oncol.

[2] Akinleye A, Chen Y, Mukhi N, Song Y, Liu D (2013). Ibrutinib and novel BTK inhibitors in clinical development. J Hematol Oncol.

[3] Akinleye A, Furqan M, Mukhi N, Ravella P, Liu D (2013). MEK and the inhibitors: from bench to bedside. J Hematol Oncol.

[4] Furqan M, Akinleye A, Mukhi N, Mittal V, Chen Y, Liu D (2013). STAT inhibitors for cancer therapy. J Hematol Oncol.

[5] Furqan M, Mukhi N, Lee B, Liu D (2013). Dysregulation of JAK-STAT pathway in hematological malignancies and JAK inhibitors for clinical application. Biomarker Res.

[6] Huang T, Karsy M, Zhuge J, Zhong M, Liu D (2013). B-Raf and the inhibitors: from bench to bedside. J Hematol Oncol.

[7] Saha M, Qiu L, Chang H (2013). Targeting p53 by small molecules in hematological malignancies. J Hematol Oncol.

[8] Ansell SM, Lesokhin AM, Borrello I, Halwani A, Scott EC, Gutierrez M, Schuster SJ, Millenson MM, Cattry D, Freeman GJ, Rodig SJ, Chapuy B, Ligon AH, Zhu L, Grosso JF, Kim SY, Timmerman JM, Shipp MA, Armand P (2015). PD-1 blockade with nivolumab in relapsed or refractory Hodgkin’s lymphoma. N Engl J Med.

[9] Brahmer J, Reckamp KL, Baas P, Crino L, Eberhardt WE, Poddubskaya E, Antonia S, Pluzanski A, Vokes EE, Holgado E, Waterhouse D, Ready N, Gainor J, Aren Frontera O, Havel L, Steins M, Garassino MC, Aerts JG, Domine M, Paz-Ares L, Reck M, Baudelet C, Harbison CT, Lestini B, Spigel DR (2015). Nivolumab versus docetaxel in advanced squamous-cell non-small-cell lung cancer. The New Eng J Med.

[10] Garon EB, Rizvi NA, Hui R, Leighl N, Balmanoukian AS, Eder JP, Patnaik A, Aggarwal C, Gubens M, Horn L, Carcereny E, Ahn MJ, Felip E, Lee JS, Hellmann MD, Hamid O, Goldman JW, Soria JC, Dolled-Filhart M, Rutledge RZ, Zhang J, Lunceford JK, Rangwala R, Lubiniecki GM, Roach C, Emancipator K, Gandhi L, Investigators K (2015). Pembrolizumab for the treatment of non-small-cell lung cancer. N Engl J Med.

[11] Larkin J, Chiarion-Sileni V, Gonzalez R, Grob JJ, Cowey CL, Lao CD, Schadendorf D, Dummer R, Smylie M, Rutkowski P, Ferrucci PF, Hill A, Wagstaff J, Carlino MS, Haanen JB, Maio M, Marquez-Rodas I, McArthur GA, Ascierto PA, Long GV, Callahan MK, Postow MA, Grossmann K, Sznol M, Dreno B, Bastholt L, Yang A, Rollin LM, Horak C, Hodi FS (2015). Combined nivolumab and ipilimumab or monotherapy in untreated melanoma. N Engl J Med.

[12] Postow MA, Chesney J, Pavlick AC, Robert C, Grossmann K, McDermott D, Linette GP, Meyer N, Giguere JK, Agarwala SS, Shaheen M, Ernstoff MS, Minor D, Salama AK, Taylor M, Ott PA, Rollin LM, Horak C, Gagnier P, Wolchok JD, Hodi FS (2015). Nivolumab and ipilimumab versus ipilimumab in untreated melanoma. N Engl J Med.

[13] Shi L, Chen S, Yang L, Li Y (2013). The role of PD-1 and PD-L1 in T-cell immune suppression in patients with hematological malignancies. J Hematol Oncol.

[14] Goede V, Fischer K, Busch R, Engelke A, Eichhorst B, Wendtner CM, Chagorova T, de la Serna J, Dilhuydy MS, Illmer T, Opat S, Owen CJ, Samoylova O, Kreuzer KA, Stilgenbauer S, Dohner H, Langerak AW, Ritgen M, Kneba M, Asikanius E, Humphrey K, Wenger M, Hallek M (2014). Obinutuzumab plus chlorambucil in patients with CLL and coexisting conditions. N Engl J Med.

[15] Younes A, Bartlett NL, Leonard JP, Kennedy DA, Lynch CM, Sievers EL, Forero-Torres A (2010). Brentuximab vedotin (SGN-35) for relapsed CD30-positive lymphomas. N Engl J Med.

[16] Palumbo A, Hajek R, Delforge M, Kropff M, Petrucci MT, Catalano J, Gisslinger H, Wiktor-Jedrzejczak W, Zodelava M, Weisel K, Cascavilla N, Iosava G, Cavo M, Kloczko J, Blade J, Beksac M, Spicka I, Plesner T, Radke J, Langer C, Ben Yehuda D, Corso A, Herbein L, Yu Z, Mei J, Jacques C, Dimopoulos MA, Investigators MM (2012). Continuous lenalidomide treatment for newly diagnosed multiple myeloma. N Engl J Med.

[17] Postow MA, Callahan MK, Wolchok JD (2015). Immune checkpoint blockade in cancer therapy. J Clin Oncol.

[18] Han E, Li X-l, Wang C-R, Li T-F, Han S-Y (2013). Chimeric antigen receptor-engineered T cells for cancer immunotherapy: progress and challenges. J Hematol Oncol.

[19] Topp MS, Gokbuget N, Stein AS, Zugmaier G, O'Brien S, Bargou RC, Dombret H, Fielding AK, Heffner L, Larson RA, Neumann S, Foa R, Litzow M, Ribera JM, Rambaldi A, Schiller G, Bruggemann M, Horst HA, Holland C, Jia C, Maniar T, Huber B, Nagorsen D, Forman SJ, Kantarjian HM (2015). Safety and activity of blinatumomab for adult patients with relapsed or refractory B-precursor acute lymphoblastic leukaemia: a multicentre, single-arm, phase 2 study. Lancet Oncol.

[20] Reusch U, Duell J, Ellwanger K, Herbrecht C, Knackmuss SH, Fucek I, Eser M, McAleese F, Molkenthin V, Gall FL, Topp M, Little M, Zhukovsky EA (2015). A tetravalent bispecific TandAb (CD19/CD3), AFM11, efficiently recruits T cells for the potent lysis of CD19(+) tumor cells. MAbs.

[21] Novero A, Ravella P, Chen Y, Dous G, Liu D (2014). Ibrutinib for B cell malignancies. Exp Hematol Oncol.

[22] Rothe A, Sasse S, Topp MS, Eichenauer DA, Hummel H, Reiners KS, Dietlein M, Kuhnert G, Kessler J, Buerkle C, Ravic M, Knackmuss S, Marschner J-P, Pogge Von Strandmann E, Borchmann P, Engert A (2015). A phase 1 study of the bispecific anti-CD30/CD16A antibody construct AFM13 in patients with relapsed or refractory Hodgkin lymphoma. Blood.

[23] Mandelboim O, Malik P, Davis DM, Jo CH, Boyson JE, Strominger JL (1999). Human CD16 as a lysis receptor mediating direct natural killer cell cytotoxicity. Proc Natl Acad Sci U S A.

[24] Hombach A, Jung W, Pohl C, Renner C, Sahin U, Schmits R, Wolf J, Kapp U, Diehl V, Pfreundschuh M (1993). A CD16/CD30 bispecific monoclonal antibody induces lysis of Hodgkin’s cells by unstimulated natural killer cells in vitro and in vivo. Int J Cancer.

[25] Hartmann F, Renner C, Jung W, da Costa L, Tembrink S, Held G, Sek A, Konig J, Bauer S, Kloft M, Pfreundschuh M (2001). Anti-CD16/CD30 bispecific antibody treatment for Hodgkin’s disease: role of infusion schedule and costimulation with cytokines. Clinical Cancer Res.

[26] Hartmann F, Renner C, Jung W, Deisting C, Juwana M, Eichentopf B, Kloft M, Pfreundschuh M (1997). Treatment of refractory Hodgkin’s disease with an anti-CD16/CD30 bispecific antibody. Blood.

[27] Reusch U, Burkhardt C, Fucek I, Le Gall F, Le Gall M, Hoffmann K, Knackmuss SH, Kiprijanov S, Little M, Zhukovsky EA (2014). A novel tetravalent bispecific TandAb (CD30/CD16A) efficiently recruits NK cells for the lysis of CD30+ tumor cells. MAbs.

[28] Holliger P, Prospero T, Winter G (1993). “Diabodies”: small bivalent and bispecific antibody fragments. Proc Natl Acad Sci U S A.

[29] Wu J, J Fu, M Zhang, Liu D: Blinatumomab: a bispecific T cell engager (BiTE) antibody against CD19/CD3 for refractory acute lymphoid leukemia. J Hematol Oncol 2015, 8:in press. 10.1186/s13045-015-0195-4 PMC4558758 26337639

[30] Asano R, Shimomura I, Konno S, Ito A, Masakari Y, Orimo R, Taki S, Arai K, Ogata H, Okada M, Furumoto S, Onitsuka M, Omasa T, Hayashi H, Katayose Y, Unno M, Kudo T, Umetsu M, Kumagai I (2014). Rearranging the domain order of a diabody-based IgG-like bispecific antibody enhances its antitumor activity and improves its degradation resistance and pharmacokinetics. MAbs.

